# Pharmacological inhibition of FOXO1 promotes lymphatic valve growth in a congenital lymphedema mouse model

**DOI:** 10.3389/fcell.2022.1024628

**Published:** 2023-01-05

**Authors:** Ololade Ogunsina, Richa Banerjee, Luz A. Knauer, Ying Yang

**Affiliations:** Department of Molecular Pharmacology & Physiology, Morsani College of Medicine, University of South Florida, Tampa, FL, United States

**Keywords:** lymphatic valve, lymphedema, FOXO transcription factors, beta-catenin, vascular biology, AS1842856, inhibition of FOXO1

## Abstract

Mutations in many genes that regulate lymphatic valve development are associated with congenital lymphedema. Oscillatory shear stress (OSS) from lymph provides constant signals for the growth and maintenance of valve cells throughout life. The expression of valve-forming genes in lymphatic endothelial cells (LECs) is upregulated by OSS. The transcription factor FOXO1 represses lymphatic valve formation by inhibiting the expression of these genes, which makes FOXO1 a potential target for treating lymphedema. Here, we tested the ability of a FOXO1 inhibitor, AS1842856, to induce the formation of new lymphatic valves. Our quantitative RT-PCR and Western blot data showed that treatment of cultured human LECs with AS1842856 for 48 h significantly increased the expression levels of valve-forming genes. To investigate the function of AS1842856 *in vivo*, *Foxc2* ^*+/−*^ mice, the mouse model for lymphedema-distichiasis, were injected with AS1842856 for 2 weeks. The valve number in AS-treated *Foxc2^+/−^* mice was significantly higher than that of the vehicle-treated *Foxc2^+/−^* mice. Furthermore, since β-catenin upregulates the expression of Foxc2 and Prox1 during lymphatic valve formation, and AS1842856 treatment increased the level of active β-catenin in both cultured human LECs and in mouse mesenteric LECs *in vivo*, we used the mouse model with constitutive active β-catenin to rescue loss of lymphatic valves in *Foxc2* ^*+/−*^ mice. *Foxc2* ^*+/−*^ mice have 50% fewer lymphatic valves than control, and rescue experiments showed that the valve number was completely restored to the control level upon nuclear β-catenin activation. These findings indicate that pharmacological inhibition of FOXO1 can be explored as a viable strategy to resolve valve defects in congenital lymphedema.

## Introduction

The lymphatic vasculature is essential for the absorption of interstitial fluids to form lymph and for the transportation of lymph back to the blood vasculature. The lymphatic capillaries absorb interstitial fluids, and the collecting lymphatic vessels transport lymph (Yang and Oliver, 2014; Breslin et al., 2018). To efficiently transport lymph against the hydrostatic pressure, the collecting lymphatic vessels pump to move lymph forward through perivascular smooth muscle cell contraction. Lymphatic valves in the collecting lymphatic vessels act as gates and open when the pressure builds up downstream of the valve leaflets to let lymph pass. Valves close when the upstream pressure of the valve leaflets is higher than the downstream pressure to ensure the forward movement of lymph in one direction (Davis et al., 2011). This unidirectional lymph flow is important for the function of the lymphatic vasculature, which is maintained by lymphatic valves. The retrograde lymph flow is one of the diagnostic characteristics of the lymphatic disease, lymphedema. Mutations in many valve genes have been identified to be a causative factor for congenital lymphedema in human patients, and the corresponding knockout mouse models of these genes have shown defects in lymphatic valve formation. For example, heterozygosity of the *FOXC2* gene causes lymphedema-distichiasis syndrome in human patients (Kriederman et al., 2003). *Foxc2* is a forkhead box, winged-helix transcription factor which regulates the maturation of collecting lymphatic vessels. During embryonic development, no lymphatic valves form in *Foxc2* knockout mice (Norrmen et al., 2009). Moreover, *Foxc2* is crucial for maintaining valves throughout life. Postnatal deletion of *Foxc2* results in loss of lymphatic valves after the valve structure has formed (Sabine et al., 2015). In the *Foxc2* heterozygous mouse model, the number of lymphatic valves is reduced by nearly 50% in the skin and the mesentery (Kanady et al., 2015; Scallan et al., 2021). The function of the remaining valves in *Foxc2* heterozygous mice is severely compromised (Castorena-Gonzalez et al., 2020; Scallan et al., 2021). Therefore, the *Foxc2* heterozygous mice are a model for human autosomal dominant lymphedema-distichiasis syndrome, demonstrating the defects in lymphatic valves.

The formation of lymphatic valves requires oscillatory shear stress (OSS) from lymph flow. VE-cadherin, an adherens jucntion protein, on the membrane functions as a key component in the mechanotransduction signaling pathway to transmit the external OSS signal into the lymphatic endothelial cells (LECs) through cytoplasmic AKT activation (Yang et al., 2019). The OSS signaling upregulates the expression of three key valve transcription factors, *Prox1*, *Foxc2*, and *Gata2*, to initiate the specification of lymphatic valve cells (Sweet et al., 2015). Similar to *FOXC2*, *GATA2* is required for lymphatic valve formation and maintenance (Kazenwadel et al., 2015). Mutations in *GATA2* cause primary lymphedema in human patients (Ostergaard et al., 2011; Kazenwadel et al., 2012). The activation of these valve-forming genes is regulated by the transcription factors, β-catenin and FOXO1, which are downstream targets in the OSS-AKT signaling pathway. β-Catenin binds to the promoter regions of *PROX1* and *FOXC2*, which activates the expression of these genes. Loss of *β-catenin* in LECs leads to defects in valve formation during embryonic development (Cha et al., 2016). In contrast, *Foxo1* is a repressor of lymphatic valve formation. When FOXO1 is in the nucleus, it binds to the promoter area of *FOXC2* to repress its expression (Scallan et al., 2021). However, when activated AKT phosphorylates FOXO1, it no longer stays in the nucleus and is degraded in the cytoplasm. Therefore, deletion of *Foxo1* in the lymphatics results in increased valve formation in healthy mice. Moreover, *Foxo1* ablation in *Foxc2* heterozygous mice, the model of human lymphedema-distichiasis disease, rescues both the valve loss and valve dysfunction to the wildtype level (Scallan et al., 2021). In this study, we investigated the effect of the pharmacological inhibition of FOXO1 using a specific inhibitor AS1842856 in cultured human dermal lymphatic endothelial cells (hdLECs) and in mice. Our data showed that hdLECs treated with AS1842856 had increased expression levels of valve-forming genes, including FOXC2, GATA2, KLF4, ITGA9, and GJA4, on both mRNA and protein levels. Intriguingly, more β-catenin was accumulated in the nucleus of hdLECs treated with AS1842856, compared to the vehicle control, which stimulates valve-forming gene expression. Furthermore, genetic activation of β-catenin in the nucleus rescued the valve loss in *Foxc2* heterozygous mice to the wildtype level. Meanwhile, treatment of AS1842856 in *Foxc2* heterozygous mice significantly increased the number of valves. Therefore, in addition to genetic deletion of *Foxo1*, valve defects in a primary lymphedema mouse model can also be rescued by using a drug to pharmacologically inhibit FOXO1.

## Results

### AS1842856 treatment results in the upregulation of valve-forming genes in a time- and concentration-dependent manner

**FIGURE 1:**
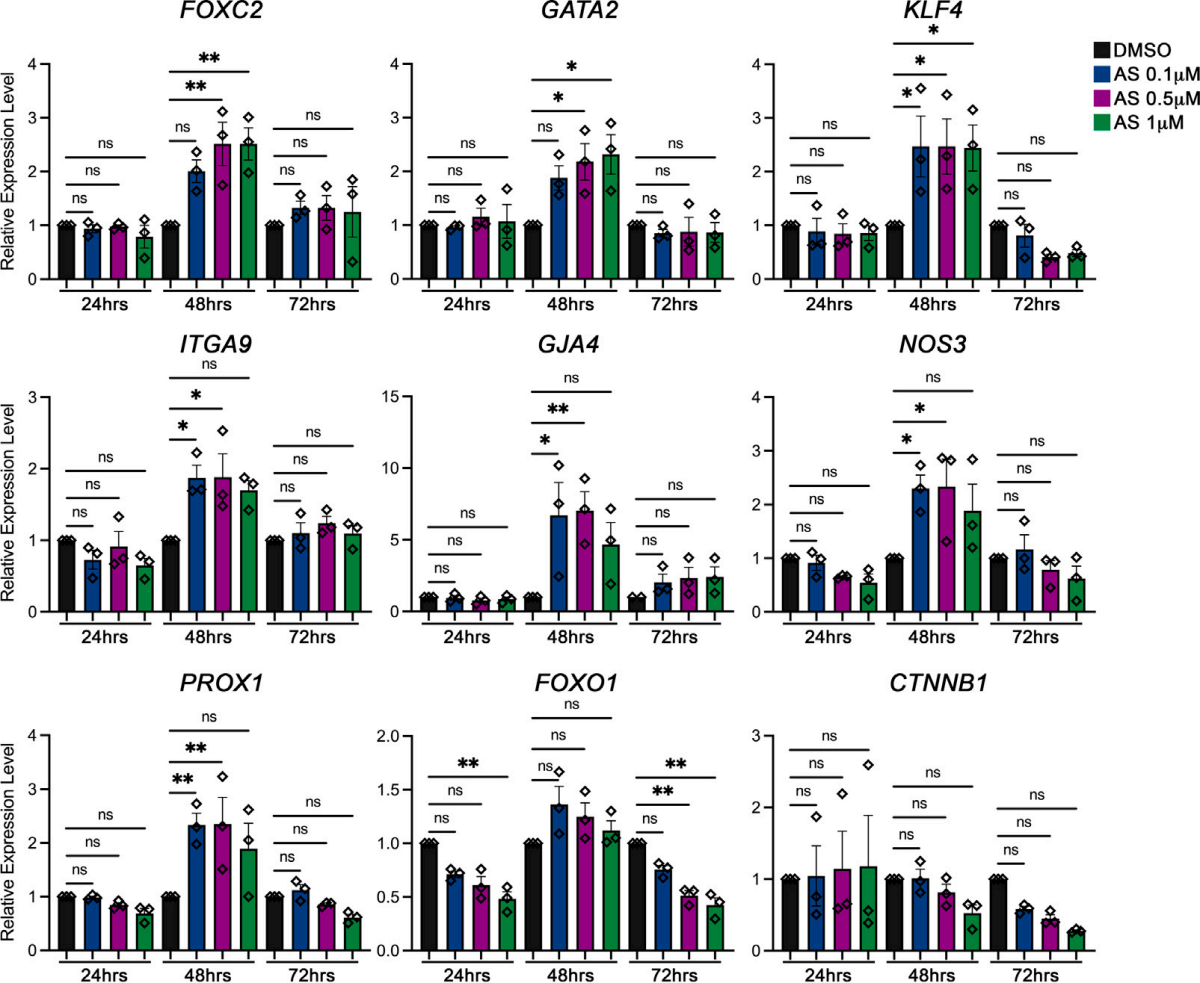
AS1842856 (AS) has a time- and concentration-dependent effect on valve-forming gene expression. Human dermal lymphatic endothelial cells (hdLECs) were cultured under static conditions and treated for the indicated time (24 h, 48 h, and 72 h) and concentrations: DMSO 1 µM (black), AS 0.1 µM (blue), AS 0.5 µM (purple), and AS 1 µM (green). Afterward, mRNA was extracted and reverse transcribed. The expression levels of indicated genes were quantified by qRT-PCR analysis. All values are mean ± SEM. *n* = 3 experiment repeats; ns = not significant; *p ≤ 0.05, **p ≤ 0.01.

The transcription factor *Foxo1* is expressed in many cell types in the body, including lymphatic endothelial cells (LECs). We have previously reported that FOXO1 acts as a repressor for lymphatic valve formation. Deletion of *Foxo1* in LECs induces the growth of new valves *in vivo*. More importantly, ablation of *Foxo1* in a mouse model for human disease lymphedema–distichiasis, the *Foxc2* heterozygous mice, completely resolves both the loss of valves and the leakiness of valves in this disease model. Mechanistically, knockdown of *FOXO1* using shRNA in human dermal LECs (hdLECs) markedly upregulates the expression of valve-forming genes, including FOXC2, GATA2, and KLF4. Furthermore, FOXO1 mediates the repression activity by directly binding to the promoter region of the *FOXC2* gene and negatively regulates *FOXC2* expression (Scallan et al., 2021). These data strongly indicate that *FOXO1* is a potential target for treating lymphedema–distichiasis. AS1842865 is a cell-permeable inhibitor of FOXO1 transcription activity and has a minimum effect on inhibiting the activity of FOXO3 and FOXO4 (Nagashima et al., 2010). Several research groups have reported that the function of FOXO1 can be pharmacologically inhibited by AS1842865 in different cell types (Zou et al., 2014; Jeon et al., 2018; Wang et al., 2020; Jeon et al., 2021). Therefore, we hypothesize that AS1842865 treatment will inhibit FOXO1 activity and upregulate the expression of valve-forming genes in hdLECs. To investigate this, hdLECs were treated with three distinct concentrations of AS1842865—0.1 µM, 0.5 µM, and 1 µM for 24 hours (h), 48 h, and 72 h, respectively. DMSO was used as the vehicle control. The medium was changed every day with the freshly prepared drug. Thereafter, the expression levels of valve-forming genes and flow-responsive genes, including *FOXC2*, *GATA2*, *PROX1*, *KLF4*, *ITGA9*, *GJA4*, *NOS3*, and *CTNNB1*, were determined using quantitative RT-qPCR. Specifically, transcription factors *FOXC2*, *GATA2*, and *CTNNB1* (*β-catenin*) have been shown to be essential for lymphatic valve formation. Deletion of any of these three genes leads to the absence of lymphatic valve formation during embryonic development (Petrova et al., 2004; Kazenwadel et al., 2015; Cha et al., 2016). Our quantitative RT-PCR results showed that the expression levels of *FOXC2* and *GATA2* were significantly upregulated with 48 h AS treatment, but not with 24 h or 72 h treatment (Figure 1). However, the expression of *CTNNB1* was not altered*.* Meanwhile, the expression levels of the key flow-responsive gene *KLF4* and the master LEC gene *PROX1* were significantly increased under the same condition. Additionally, it is reported that both ITGA9 (integrin α9) and GJA4 (connexin37) are highly expressed in the valve leaflets. Loss-of-function of either gene results in defective valves (Bazigou et al., 2009; Kanady et al., 2011). Our data showed that AS treatment for 48 h induced significantly higher expression levels of *ITGA9* and *GJA4* (Figure 1). NOS3 (eNOS) is a downstream gene in the mechanotransduction pathway in blood endothelial cells (BECs), which is expressed in lymphatic valve cells and is regulated by FOXC2 in LECs (Sabine et al., 2012). Our results showed that *NOS3* expression was significantly increased with 48 h AS treatment as well. Furthermore, as expected, the expression level of FOXO1 was not altered by AS treatment because AS1842865 does not affect either the transcription or the translation of FOXO1. Instead, AS1842865 binds to the FOXO1 protein to prevent the binding of FOXO1 to the promoter regions and reduces the transcription activity of FOXO1 in the nucleus (Nagashima et al., 2010). In summary, the expression pattern of valve-forming genes with respect to time showed a significant increase at 48 h treatment of AS1842865, but not at 24 h or 72 h (Figure 1).

In addition to testing the length of the treatment, three concentrations of AS1842865, 0.1 µM, 0.5 µM, and 1 µM, were used. Since we had revealed that 48 h treatment was the only time point to show the expression changes in valve-forming genes, we focused on discussing the results from 48 h AS treatment from that point on. Although the expression levels of *FOXC2* and *GATA2* displayed a trend of increase with 0.1 µM treatment, both 0.5 µM and 1 µM AS treatment induced a significant increase in the expression of *FOXC2* and *GATA2*. Meanwhile, *KLF4* expression was increased by the treatment of all three concentrations of AS. Furthermore, the expression levels of *ITGA9*, *GJA4*, *NOS3*, and *PROX1* were upregulated only under 0.1 µM and 0.5 µM, but not under 1 µM. None of the concentrations induced significant upregulation of either *FOXO1* or *CTNNB1* expression (Figure 1). Overall, the quantitative RT-PCR results showed that the FOXO1 inhibitor AS1842865 induced the expression of valve-forming genes in a time- and concentration-dependent manner. We showed that 0.5 µM of AS1842865 treatment for 48 h provided the best results for the increased expression of *FOXC2*, *GATA2*, *KLF4*, *ITGA9*, *GJA4*, *NOS3*, and *PROX1*.

### AS1842856 treatment increases the nuclear activity of β-catenin in hdLECs

**FIGURE 2:**
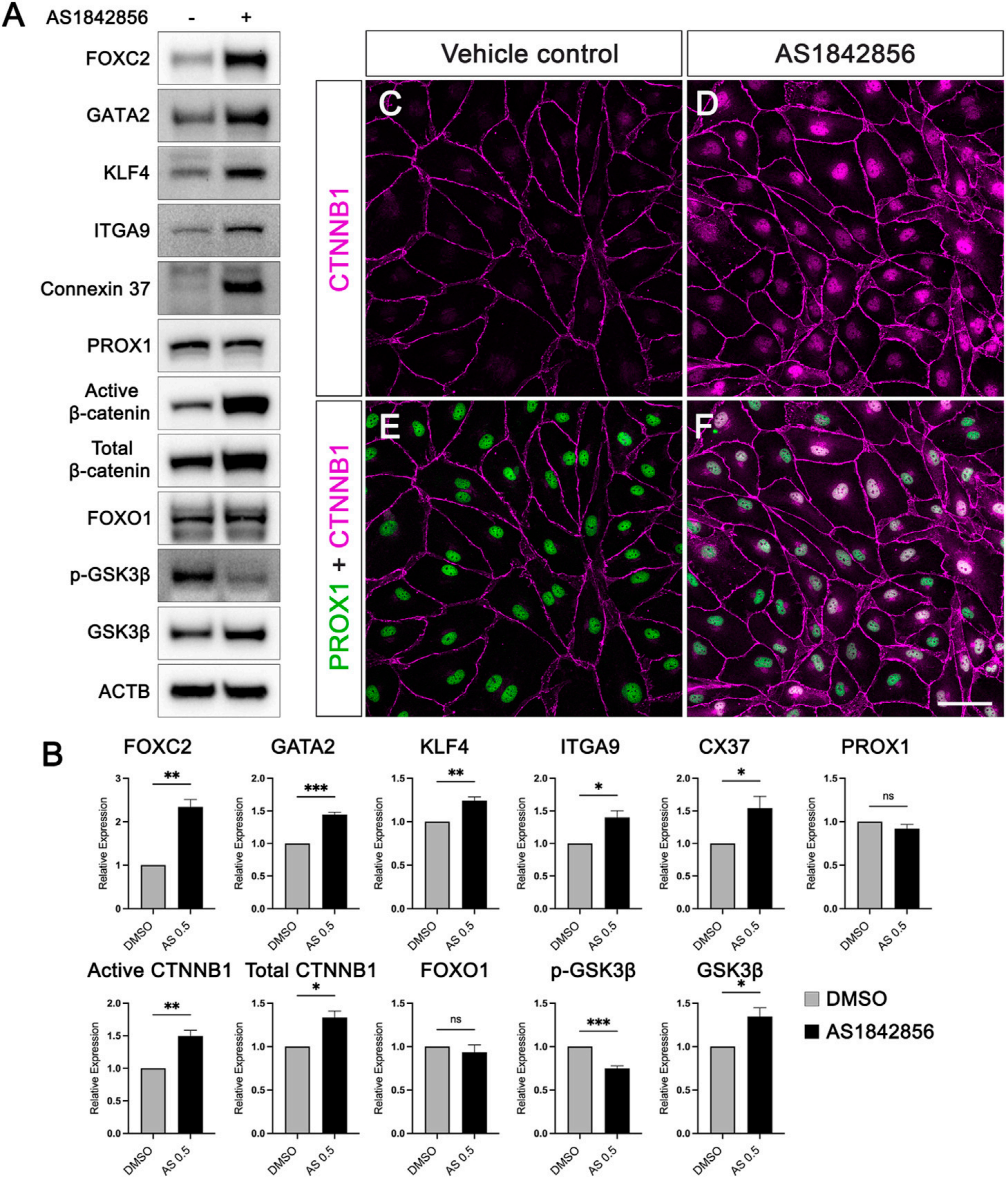
AS1842856 elevates protein expression of valve-forming genes and promotes β-catenin nuclear localization in cultured hdLECs. **(A)** hdLECs were cultured under static conditions and treated for 48 h with 0.5 µM DMSO (vehicle control) or 0.5 µM AS treatment. Thereafter, proteins were extracted, and the Western blot was performed for the indicated proteins. ACTB is the loading control. **(B)** Quantification results of indicated proteins in the Western blot from three independent experiments. **(C–F)** Immunostaining of cultured hdLECs for *PROX1* and CTNNB1 proteins. All values are mean ± SEM. *n* = 3 experiment repeats; ns = not significant; *p ≤ 0.05, **p ≤ 0.01, ***p ≤ 0.001. Vehicle control: 0.5 µM DMSO for 48 h treatment. AS1842856: 0.5 µM AS1842856 for 48 h treatment. The cells were stained for *PROX1* (green) and CTNNB1 (purple). **(E, F)** Merged images for *PROX1* and CTNNB1. Scale bar is 50 μm.

We have identified that 0.5 µM of AS1842865 treatment for 48 h significantly increased the expression levels of valve-forming genes on the mRNA level. Next, we performed Western blot on the cells treated with 0.5 µM for 48 h to determine whether the expression of these genes on the protein level was consistent with the PCR results. Similar to the quantitative RT-qPCR, three independent experiments of cultured hdLECs were performed. Protein levels of FOXC2, GATA2, KLF4, ITGα9, GJA4, active β-catenin, total β-catenin, PROX1, and FOXO1 were quantified from the Western blot. As expected, the protein expression levels of FOXC2, GATA2, KLF4, ITGA9, and GJA4 were significantly upregulated upon AS treatment compared to the vehicle control, while FOXO1 protein expression did not change in response to AS treatment, which was consistent with the results from their mRNA expression (Figure 2B). However, the protein level of PROX1 did not alter although the mRNA level of PROX1 was increased. Notably, the amount of active β-catenin and total β-catenin protein increased significantly in AS-treated cells (Figure 2B). Since the active β-catenin antibody only detects the non-phosphorylated form of β-catenin, which is the pool of β-catenin that is not phosphorylated by glycogen synthase kinase 3beta (GSK3β, one of the key components of the destruction complex) to be degraded by the destruction complex, our Western blot results suggested that AS1842865 treatment could inhibit the degradation of β-catenin by reducing β-catenin phosphorylation to increase the amount of non-phosphorylated β-catenin. Intriguingly, the protein amount of total β-catenin also increased in the AS treatment despite the unchanged mRNA level of β-catenin under the same condition. This further confirmed that AS1842865 regulated the amount of β-catenin on a post-transcriptional level. To investigate whether the activity of the β-catenin destruction complex was decreased in response to AS1842865 treatment, the levels of GSK3β and its phosphorylated form (i.e., the inactive form) p-GSK3β were analyzed in the Western blot. Unexpectedly, the amount of total GSK3β was significantly increased and the amount of p-GSK3β was significantly decreased (Figure 2B). These results indicate that AS1842865 treatment increases the nuclear activity of β-catenin through different mechanisms other than inhibiting the destruction complex through GSK3β.

In cells, phosphorylated β-catenin is degraded in a destruction complex in the cytoplasm, but unphosphorylated β-catenin upon activation is translocated to the nucleus from the cytoplasm (Harada et al., 1999). It has been reported that β-catenin in the nucleus of LECs promotes the expression of valve-forming genes *FOXC2* and *PROX1* by binding to the promoter areas of these two genes (Cha et al., 2016). Since we observed that AS1842865 treatment increased the amount of active β-catenin (non-phosphorylated) in the Western blot (Figure 2A), we decided to perform immunostaining of the LEC transcription factor PROX1 and β-catenin in hdLECs treated with 0.5 µM AS for 48 h to visualize the localization of β-catenin. Under the control condition (DMSO), β-catenin was almost exclusively localized on the cell membrane in the vehicle-treated hdLECs, while PROX1 is only in the nucleus (Figures 2C,E). Strikingly, a remarkable amount of β-catenin was in the nucleus of AS-treated cells in addition to the cell membrane (Figure 2D). Merged confocal images showed the co-localization of PROX1 and β-catenin in the nucleus of AS-treated cells (Figure 2F). These data indicated that 1) the nuclear β-catenin observed in immunostaining represented the non-phosphorylated β-catenin shown in the Western blot, and 2) AS1842865 treatment increased the nuclear activity of β-catenin in LECs, which would upregulate the expression of valve-forming genes, such as *FOXC2*.

Collectively, we have shown that AS1842856 increased the expression of valve-forming genes at the mRNA and protein levels in a concentration- and time-dependent manner in cultured hdLECs by inhibiting FOXO1 activity and by increasing β-catenin activity in the nucleus.

### AS1842856 treatment increases the valve number in *Foxc2* heterozygous mice

**FIGURE 3:**
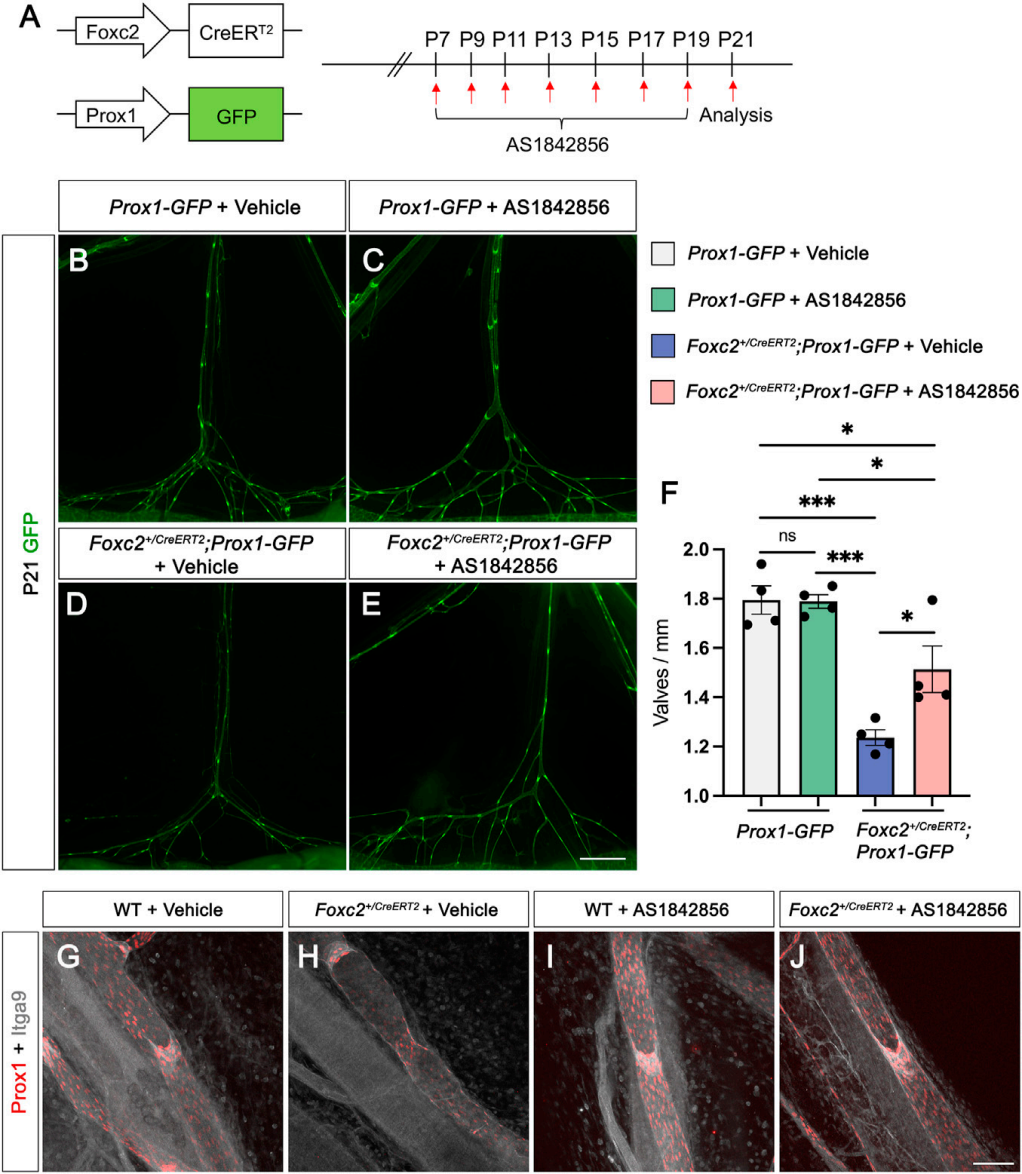
AS1842856 treatment *in vivo* increases the valve number in *Foxc2* ^*+/CreERT2*^ mice. **(A)** Schematic illustration of AS1842856 injection procedure in postnatal day (P) 7–21 pups. **(B–E)** Fluorescent images show the morphology of mesenteric collecting lymphatic vessels at P21. Mesenteries are from *Prox1-GFP* with DMSO (vehicle control) injection **(B)**; *Prox1-GFP* with AS1842856 injection **(C)**; *Foxc2* ^*+/CreERT2*^; *Prox1-GFP* with DMSO injection **(D)**; *Foxc2* ^*+/CreERT2*^; *Prox1-GFP* with AS1842856 injection **(E)**. **(F)** Quantification of valves per millimeter at P21 from four groups of mice. A total of four mice were used for the analysis. **(G–J)** Whole immunostaining of P14 mesenteries from WT with DMSO (vehicle control, G); *Foxc2^+CreERT2^* with DMSO **(H)**; WT with AS1842856 injection **(I)**; *Foxc2^+CreERT2^* with AS1842856 injection **(J)** with Prox1 (Red) and Itga9 (gray). All values are mean ± SEM. Two-way ANOVA was performed with Tukey’s multiple-comparison test. ns = not significant, *p ≤ 0.05, ***p < 0.001. Scale bar is 1,000 μm for **(B–E)** and is 100 μm for **(G-J)**.

**FIGURE 4:**
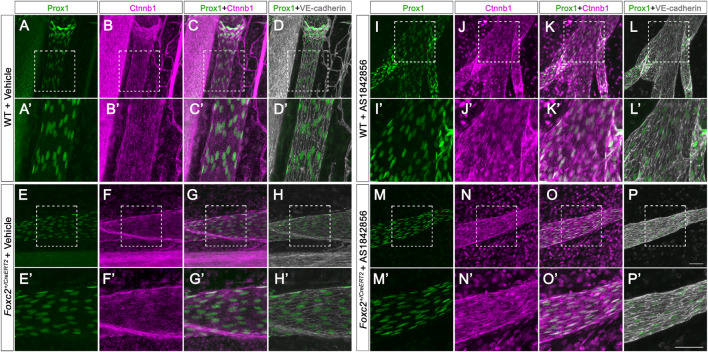
AS1842856 injection promotes β-catenin nuclear localization *in vivo*. **(A–P′)** Whole-mount immunostaining of P11 mesenteric collecting lymphatic vessels from wildtype and *Foxc2* ^*+/CreERT2*^ pups with vehicle control (DMSO) or AS1842856 injection. *Prox1* is shown in green, Ctnnb1 in purple, and VE-cadherin in gray. **(A′–P′)** shows higher magnification images of the white box areas **(A–P)**. Scale bars are 100 μm.

*Foxc2* is a chief regulator of lymphatic valve development modulating the expression of many valve genes (Sabine et al., 2015). Although *Foxc2*-null animals are embryonically lethal, the *Foxc2* heterozygous mice are a model of the human disease lymphedema-distichiasis with severe lymphatic valve loss (Kriederman et al., 2003; Petrova et al., 2004). To test whether AS1842856 treatment increases valve growth *in vivo*, we intraperitoneally (i.p.) injected AS1842856 into *Foxc2* ^*+/CreERT2*^ pups. Instead of using wildtype pups as control, we used *Prox1-GFP* mice as control and for visualizing the lymphatic vasculature (Choi et al., 2011). The *Foxc2* ^*+/CreERT2*^ pups were also bred to carry the *Prox1-GFP* allele. Since the open reading frame in one allele of the *Foxc2* gene is replaced with the cDNA for CreER^T2^, the *Foxc2* ^*+/CreERT2*^ mice is heterozygous for the *Foxc2* gene (Geng et al., 2016). Four groups of pups were used: 1) *Prox1-GFP* pups with DMSO (vehicle control) injection; 2) *Prox1-GFP* pups with AS1842856 injection; 3) *Foxc2* ^*+/CreERT2*^; *Prox1-GFP* pups with DMSO injection; and 4) *Foxc2* ^*+/CreERT2*^, *Prox1-GFP* pups with AS1842856 injection. DMSO and AS1842856 injections were performed every other day from P7 to P21 (Figure 3A). To compare the number of valves from these four groups, we counted the total number of valves from six branches (from the lymph nodes to the gut wall) in the mesentery and measured the total length of these branches as we previously published (Scallan et al., 2021). The average valve number per mm (valves/mm) was used for comparison. Our quantitative results showed that 1) valves/mm was significantly lower in *Foxc2* ^*+/CreERT2*^ pups with DMSO injection than those in *Prox1-GFP* pups with DMSO injection; 2) valves/mm was significantly higher in *Foxc2* ^*+/CreERT2*^ pups with AS1842856 injection than those in *Foxc2* ^*+/CreERT2*^ pups with DMSO injection; 3) valves/mm was still significantly lower in *Foxc2* ^*+/CreERT2*^ pups with AS1842856 injection than those in *Prox1-GFP* pups with DMSO injection; and 4) there was no significant difference in valves/mm between *Prox1-GFP* pups with DMSO injection and *Prox1-GFP* pups with AS1842856 injection (Figures 3B–F). Whole-mount immunostaining of Prox1 and Itga9 was performed to visualize the morphology of valves with and without AS1842856 treatment in wildtype and *Foxc2^+/CreERT2^* pups (Figures 3G–J). Our data showed that 1% of the valve areas with high Prox1 expression in the *Foxc2* ^*+/CreERT2*^ pups did not express Itga9 (Figure 3H), which is consistent with our previous publication (Scallan et al., 2021) and suggested that these valves are not mature. However, all the valves from AS1842856-treated *Foxc2* ^*+/CreERT2*^ pups expressed Itga9. To further demonstrate the effect of AS1842856 injection on the nuclear localization of β-catenin *in vivo*, we whole-mount immunostained Prox1 in green, β-catenin in purple, and VE-cadherin in gray on the mesenteries after three injections of DMSO or AS1842856 (Figures 4A–P′). We revealed that β-catenin was mostly localized on the cell membrane in the DMSO-injected group, indicated by the strong nuclear green Prox1 staining in the Prox1- and β-catenin-merged images (Figures 4C, C′, G, G′). In contrast, due to the increased nuclear purple staining from β-catenin in the AS1842856-injected group, the nuclei became gray-colored in the Prox1 (green)- and β-catenin (purple)-merged images (Figures 4K, K′, O, O′). In conclusion, AS1842856 injection significantly increased lymphatic valves in *Foxc2* ^*+/CreERT2*^ pups but did not rescue the defect to the wildtype level.

### Increased nuclear activity of β-catenin *in vivo* restores the valve loss in *Foxc2* heterozygous mice

**FIGURE 5:**
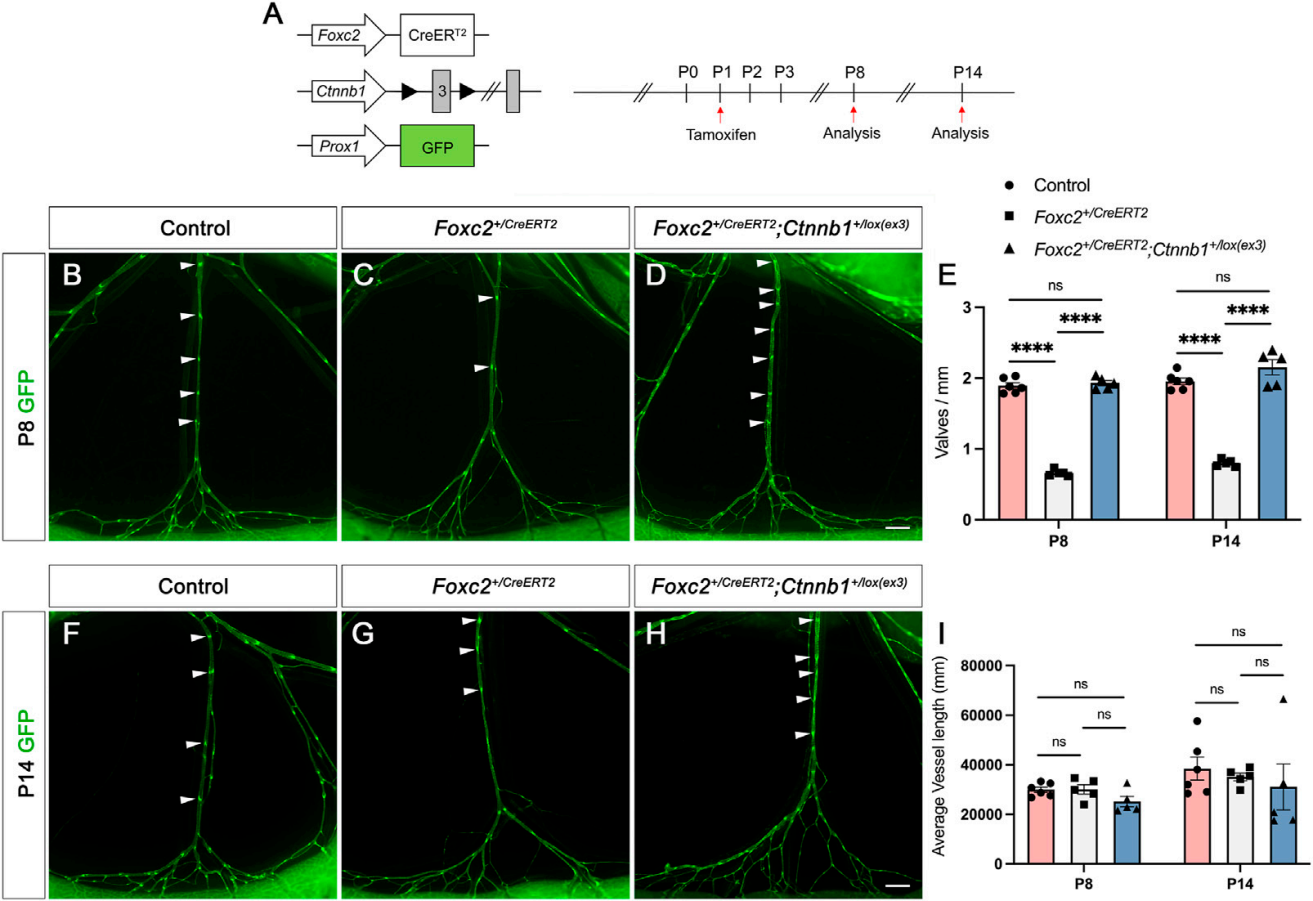
Overactivation of β-catenin in the nucleus rescues valve loss in *Foxc2* ^*+/CreERT2*^ mice. **(A)** Schematic illustration of tamoxifen injection procedure that results in deletion of exon 3 of *Ctnnb1 via CreER* ^*T2*^ activation. **(B–D)** Fluorescent images show the morphology of mesenteric collecting lymphatic vessels at postnatal day (P) 8. **(E)** Quantification of valves per millimeter at P8 and P14. **(F–H)** Fluorescent images show the morphology of mesenteric collecting lymphatic vessels at P14. **(I)** Quantification of vessel length at P8 and P14. White arrowheads point to valves determined by high GFP (green) expression. Six control, five *Foxc2* ^*+/CreERT2*^, and five *Foxc2* ^*+/creERT2*^; *Ctnnb1* ^*+/flox(exo3*)^ mesenteries are used for the analysis. All values are mean ± SEM. One-way ANOVA was performed with Tukey’s multiple-comparison test for P8 and P14, respectively. ns = not significant, ****p < 0.0001. Scale bars are 500 µM **(B,C,D,F,G,H)**.

We have discovered that AS1842856 treatment increased the nuclear activity of β-catenin in both cultured hdLECs and *in vivo*, and AS1842856 injection *in vivo* led to the growth of new valves in *Foxc2* heterozygous pups. Furthermore, it is reported that β-catenin directly binds to the *FOXC2* promoter region to activate the expression of *FOXC2* (Cha et al., 2016). It indicates that more nuclear β-catenin induced by FOXO1 inhibition could partially contribute to the growth of new valves. Therefore, we hypothesized that increased activity of β-catenin in the nucleus will restore the valve loss in *Foxc2* heterozygous mice. To increase the nuclear activity of β-catenin *in vivo*, we used the *Ctnnb1* ^*lox(ex3)*^ mice, in which the serine and threonine encoded by exon 3 of the β-catenin gene (*Ctnnb1*) and phosphorylated by GSK3β are deleted to prevent the degradation of β-catenin in the destruction complex (Harada et al., 1999). We bred the *Foxc2* ^*+/CreERT2*^ mice with the *Ctnnb1* ^*lox(ex3)*^ mice and the LEC reporter line, *Prox1-GFP*, for visualizing the lymphatic vasculature (Choi et al., 2011) to obtain the *Foxc2^+CreERT2^*; *Ctnnb1^lox(ex3)^*; *Prox1-GFP* mice. As mentioned previously, the open reading frame in one allele of the *Foxc2* gene is replaced with the cDNA for CreER^T2^ in the *Foxc2* ^*+/CreERT2*^ mice making it heterozygous for *Foxc2* and carrying tamoxifen-inducible Cre activity to recombine loxP sites. We injected tamoxifen (TM) at postnatal day (P) 1 and analyzed the mesenteric lymphatic vasculature at P8 and P14 in the *Foxc2^+/CreERT2^*; *Prox1-GFP* and *Foxc2^+/CreERT2^*; *Ctnnb1^lox(ex3)^*; *Prox1-GFP* and the control *Ctnnb1^lox(ex3)^*; *Prox1-GFP* mice (Figure 5A). The valves/mm and the average vessel length were used for comparison. The morphology of the lymphatic vasculature shown by the GFP images demonstrated that the distance between two adjacent valves was longer in the *Foxc2* ^*+/CreERT2*^; *Prox1-GFP* mesentery than that in either the control or the *Foxc2* ^*+/CreERT2*^; *Ctnnb1* ^*lox(ex3)*^; *Prox1-GFP* mice (Figures 5B–D). To support this finding, our quantitative data confirmed that valves/mm was significantly reduced in *Foxc2* ^*+/CreERT2*^; *Prox1-GFP* mice at P8 compared to the control mesentery (Figure 5E). The vessel length was not significantly different between the three genotypes (Figure 5I). Remarkably, the valve loss in the *Foxc2* ^*+/CreERT2*^; *Prox1-GFP* mice was completely rescued by the increased nuclear activity of β-catenin in the *Foxc2* ^*+/CreERT2*^; *Ctnnb1* ^*lox(ex3)*^; *Prox1-GFP* mice (Figure 5E). Moreover, this rescue phenotype was consistent in the P14 mesentery. The significantly reduced valves/mm in the *Foxc2* ^*+/CreERT2*^; *Prox1-GFP* mesentery were restored to control levels in the *Foxc2* ^*+/CreERT2*^; *Ctnnb1* ^*lox(ex3)*^; *Prox1-GFP* mice at P14 (Figures 5E–I). In summary, our data revealed that enhancing the activity of β-catenin in the nucleus of LECs can induce the growth of new valves, which can rescue the valve loss caused by impaired development of valves in the *Foxc2* heterozygous mice, a congenital lymphedema mouse model.

### Valves in *Foxc2* ^*+/CreERT2*^; *Ctnnb1* ^*lox(ex3)*^ mice express valve genes and exhibit normal junctions

**FIGURE 6:**
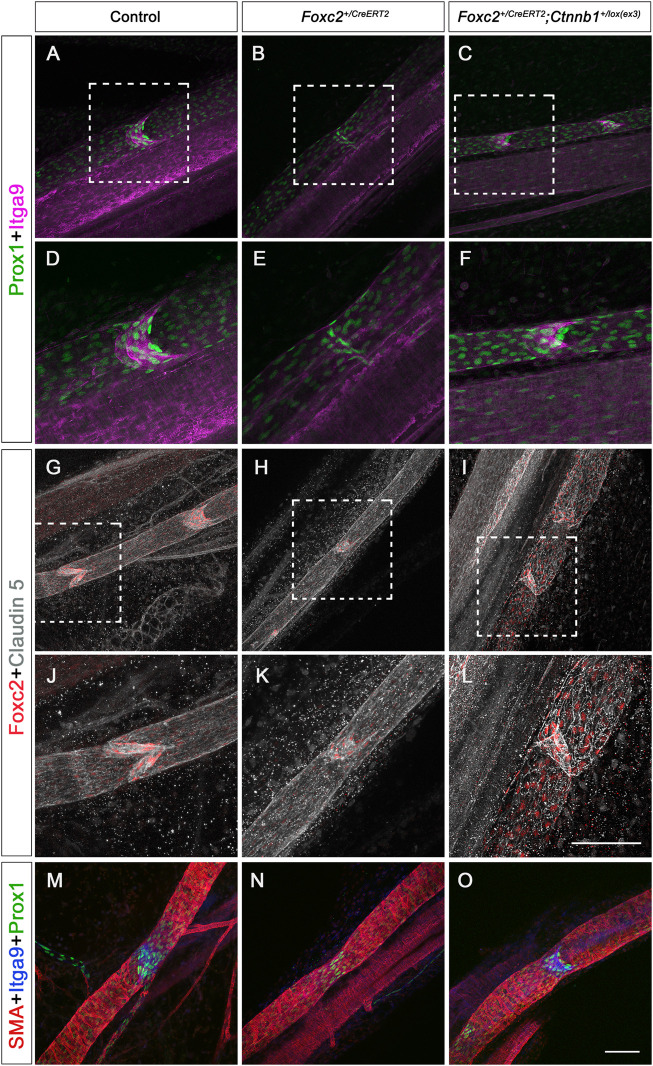
Immunostaining of valve genes, tight junction proteins, and SMA in P8 and P14 mesenteric vessels with postnatal deletion. **(A–O)** Whole-mount immunostaining of mesenteries with P1 TM injection from control, *Foxc2^+/CreERT2^*, and *Foxc2^+/CreERT2^*; *Ctnnb1^+/lox(ex3)^* with Prox1 (green), Itga9 (purple), Foxc2 (red), and claudin 5 (gray) at P8, SMA (red), Itga9 (blue), and Prox1 (green) at P14. **(D–F, J–L)** are higher magnification images of **(A,B,C,G,H,I)**. Scale bars are 100 μm. Four controls and four knockouts were used in the analysis.

We have shown that increased β-catenin in the nucleus induced lymphatic valves to grow. We wanted to know whether those valves in the *Foxc2* ^*+/CreERT2*^; *Ctnnb1* ^*lox(ex3)*^ mesentery expressed the same valve genes as the controls. The same TM protocol was performed as in Figure 4A and P8 mesentery was used for immunostaining. Since both Prox1 and Foxc2 are upregulated in the valve cells during valve formation while Itga9 is specifically expressed in the valve leaflets, whole-mount immunostaining of valve genes Prox1, Foxc2, and Itga9 was performed in P8 mesenteries from the control, the *Foxc2* ^*+/CreERT2*^, and the *Foxc2* ^*+/CreERT2*^; *Ctnnb1* ^*lox(ex3)*^ mice. Considering that we do not have a method to distinguish the pre-existing valves from the new valves induced by β-catenin, we imaged multiple areas in the mesenteric lymphatic vessels in the *Foxc2* ^*+/CreERT2*^; *Ctnnb1* ^*lox(ex3)*^ mice and compared the expression pattern of those valve genes to the control and *Foxc2* ^*+/CreERT2*^. While all the valves in both the control and the *Foxc2* ^*+/CreERT2*^; *Ctnnb1* ^*lox(ex3)*^ mesentery expressed high levels of Prox1, Foxc2, and Itga9, 1% of Prox1^high^ valve areas in *Foxc2* ^*+/CreERT2*^ mice lacked the expression of Itga9, indicating that these valve areas were missing the valve leaflets, which is consistent with our previous findings (Scallan et al., 2021) (Figures 6A–F). Therefore, our data confirmed that the loss of valve leaflets indicated by Itga9 staining in *Foxc2* ^*+/CreERT2*^ was restored by increasing nuclear activity of β-catenin. In addition to valve genes, tight junction protein, caludin 5, was immunostained. Our data showed that the collecting lymphatic vessels from all three genotypes had continuous junctions displayed by the expression of claudin 5 (Cldn5) (Figures 6G–L). Therefore, increased activity of β-catenin in the nucleus did not disrupt the formation of junctions. We did not observe lymph leakage in these animals.

Mature collecting lymphatic vessels have abundant smooth muscle cell (SMC) coverage (Oliver, 2004). Loss of SMC recruitment indicates defective formation and maturation of the collecting lymphatic vessels (Breslin et al., 2018). To test whether more β-catenin in the nucleus affects SMC recruitment, we immunostained the mesentery with smooth muscle alpha-actin (SMA) in the control, *Foxc2* ^*+/CreERT2*^, and *Foxc2* ^*+/CreERT2*^; *Ctnnb1* ^*lox(ex3)*^ mice at P14. Our SMA staining showed that SMCs were recruited to the collecting lymphatic vessels in all three genotypes, and the increased nuclear activity of β-catenin in the nucleus did not affect SMC recruitment (Figures 6M–O). Thus, the increased nuclear activity of β-catenin in LECs specifically induced the formation of lymphatic valves in the *Foxc2* heterozygous mice and rescued the valve loss in this mouse model of lymphedema-distichiasis without altering the expression of junction proteins and SMC coverage.

## Discussion

Previously, we showed that deletion of *Foxo1* in the lymphatic endothelium induces new lymphatic valves to grow, which rescues the lymphatic valve defects in the *Foxc2* heterozygous mice, a mouse model of lymphedema-distichiasis, by increasing the number of valves and by enhancing the function of valves. It was the first study to show that valve defects in congenital lymphedema can be restored after the impaired lymphatic vasculature has formed, which drove us to further investigate the function of FOXO1 inhibition in treating lymphedema. In this study, we tested the effect of a FOXO1-specific inhibitor AS1842865 in hdLECs and found that AS1842865 treatment upregulated the expression of valve and flow-responsive genes, including FOXC2, GATA2, KLF4, ITGA9, GJA4, and NOS3, on both the mRNA and protein levels. Moreover, i.p injection of AS1842865 increased the valve number in *Foxc2* heterozygous mice *in vivo*. Intriguingly, Western blot showed that AS1842865 treatment increased the level of non-phosphorylated β-catenin, the active form of β-catenin, which was visualized by immunostaining in the nucleus of both cultured hdLECs and mouse mesenteric LECs *in vivo*. These data indicated that AS1842865 treatment enhanced the nuclear activity of β-catenin in LECs. To further investigate the function of nuclear β-catenin *in vivo*, we genetically increased the activity of β-catenin in the nucleus of LECs in *Foxc2* heterozygous mice, a mouse model of lymphedema-distichiasis. Strikingly, enhanced nuclear β-catenin restored the valve loss in *Foxc2* heterozygous mice to the wildtype level. Therefore, in addition to deletion of *Foxo1*, activation of β-catenin in the nucleus of LECs induces new lymphatic valves to grow.

**TABLE 1:** Immunostaining antibodies.

Antibody	Catalog number and company	Dilution
Prox1	ab101851 (Abcam)	1:500
Prox1	AF2727 (R&D)	1:500
Cdh5	550548 (BD)	1:250
Foxc2	AF6989 (R&D)	1:500
Pecam1	550274 (BD)	1:500
Itga9	AF3827 (R&D)	1:500
FOXO1	2880 (Cell Signaling)	1:500
Cldn5	34–1600 (Thermo Fisher)	1:500
Alexa Fluor 488-conjugated GFP	A21311 (Life Technologies)	1:1,000
Alexa Fluor 488 donkey anti-rabbit IgG (H + L)	A21206 (Life Technologies)	1:300
Alexa Fluor 488 donkey anti-goat IgG (H + L)	A11055 (Life Technologies)	1:300
Alexa Fluor 488 donkey anti-sheep IgG (H + L)	A11015 (Life Technologies)	1:300
Alexa Fluor 488 donkey anti-rat IgG (H + L)	A21208 (Life Technologies)	1:300
Alexa Fluor 594 donkey anti-rabbit IgG (H + L)	A21207 (Life Technologies)	1:300
Alexa Fluor 594 donkey anti-goat IgG (H + L)	A11058 (Life Technologies)	1:300
Alexa Fluor 594 donkey anti-sheep IgG (H + L)	A11016 (Life Technologies)	1:300
Alexa Fluor 594 donkey anti-rat IgG (H + L)	A21209 (Life Technologies)	1:300
Alexa Fluor 647 donkey anti-rabbit IgG (H + L)	A-31573 (Life Technologies)	1:300
Alexa Fluor 647 donkey anti-goat IgG (H + L)	A-21447 (Life Technologies)	1:300
Alexa Fluor 647 donkey anti-rat IgG (H + L)	712–605–153 (Jackson ImmunoResearch)	1:300

**TABLE 2:** Western blot antibodies.

Antibody	Catalog number and company	Dilution
FOXC2	SC-515234 (Santa Cruz)	1:100
GATA2	AF 2046 (R&D systems)	1:100
KLF4	NBP217070 (Novus Biologicals)	1:333
ITGA9	MAB4574 (R&D systems)	1:100
Non-phospho (active) β-catenin	#8814 (Cell Signaling)	1:200
Total β-catenin	#9562 (Cell Signaling)	1:1,000
FOXO1	#2880 (Cell Signaling)	1:1,000
Connexin 37	CX37A11A (Alpha Diagnostic)	1:100
PROX1	11067-2-AP (Proteintech)	1:500
p-Gsk3β	#9336 (Cell Signaling)	1:500
Gsk3β	#12456 (Cell Signaling)	1:500
β-Actin	#4967 (Cell Signaling)	1:2000
Donkey anti-goat IgG (H + L), HRP	A16005 (Invitrogen)	1:2000
Donkey anti-rabbit IgG (H + L), HRP	A16035 (Invitrogen)	1:2000
Donkey anti-mouse IgG (H + L), HRP	A16017 (Invitrogen)	1:2000

### AS1842865 treatment increases the expression of lymphatic valve-forming genes

Lymphatic valve formation requires activated signaling from oscillatory shear stress (OSS) from lymph (Sweet et al., 2015). It has been shown that OSS treatment in LECs upregulates the expression of key valve-forming transcription factors Foxc2 and Gata2. Although the expression level of Prox1 in cultured LECs does not change in response to flow treatment, Foxc2, Prox1, and Gata2 have been described as key transcription factors *in vivo* to begin valve development, and they cooperatively regulate each other in a feed-forward mechanism (Kazenwadel et al., 2015; Sabine et al., 2015). Despite that flow treatment was not applied in our study, AS1842865 treatment alone resulted in the increase in the expression of these key lymphatic valve-forming genes, which is consistent with our previous finding that FOXO1 directly binds to the promoter region of *FOXC2* to repress *FOXC2* expression and *FOXO1* knockdown in cultured hdLECs results in the upregulation of the valve-forming genes (Scallan et al., 2021). These data indicate that the shear stress signaling pathway is activated in the LECs with AS1842865 treatment without flow, supported by the upregulation of the major flow-responsive gene *KLF4*. Additionally, many other valve genes including *ITGA9*, *GJA4*, and *NOS3* were upregulated by AS1842865 treatment. Since it has been reported that GJA4 (connexin37) expression is regulated by *Foxc2* (Sabine et al., 2012), the upregulation of GJA4 in AS1842865 treatment could be due to 1) the inhibition of FOXO1 transcription activity by AS1842865 and 2) the increased expression of FOXC2. The same mechanisms can be applied on the upregulation of NOS3, which is a downstream target of FOXC2 (Sabine et al., 2012).

Moreover, AS1842865 treatment did not upregulate the mRNA level of β-catenin but increased the nuclear localization and the activity of β-catenin on the protein level. In addition, we used *shFOXO1* to knockdown *FOXO1* in cultured hdLECs and obtained the same increase in non-phospho β-catenin in *shFOXO1* LECs compared to the cells treated with scramble control (data not shown). These unexpected results led to a question that we currently do not have an answer for: how, specifically, has AS1842865 increased the translocation of β-catenin into the nucleus? To investigate this, we examined the protein levels of GSK3β, the key component of the β-catenin destruction complex and its inactive form phospho-GSK3β in the AS1842865-treated LECs. Since our results showed that non-phospho β-catenin was increased, meaning less β-catenin was degraded in the destruction complex, we should observe an increased level of phospho-GSK3β, the inactive form of GSK3β, and decreased level of GSK3β. However, the expression level of phospho-GSK3β was decreased and the expression level of GSK3β was increased, which was the opposite to what we expected. Since the increased level of nuclear β-catenin was not due to GSK3β activity, we will analyze the activity of other components of the destruction complex and the activity of Wnt ligands in future studies. Nevertheless, the increased nuclear β-catenin added one more explanation for the upregulation of FOXC2 expression because β-catenin directly binds to the promoter region of the *FOXC2* gene to activate FOXC2 expression. In summary, AS1842865 treatment potentially triggered the activation of a signaling cascade that regulates flow response and lymphatic valve formation without the presence of flow.

### AS1842865 treatment and nuclear β-catenin activation restore the valve loss in Foxc2 heterozygous mice

*Foxc2* heterozygous mice have been characterized as the mouse model for human lymphedema-distichiasis disease (Kriederman et al., 2003). These mice on the C57B6 background lack ∼40%–50% valves in number compared to wildtype mice, and the remaining valves have functional defects with leaky valve leaflets that cannot seal against the pressure from lymph (Castorena-Gonzalez et al., 2020; Scallan et al., 2021). Previously, we showed that deletion of *Foxo1* resolves both the valve loss and the valve functional defects by inducing the growth of healthy new valves in *Foxc2* heterozygous mice (Scallan et al., 2021). Therefore, we wanted to investigate the effect of a pharmacological method in inducing valve growth in this study. Our data showed that AS1842865 treatment significantly increased valve growth in *Foxc2* heterozygous mice. Unlike genetic deletion of *Foxo1*, AS1842865 treatment neither rescued the valve loss in *Foxc2* heterozygous mice to the wildtype level nor increased the valve number in wildtype mice. We suspected that this could be due to the low efficiency of transient Foxo1 inhibition by AS1842865 *in vivo* through i.p. injection, which is supported by our previous data that one allele deletion of *Foxo1* did not resolve the valve loss in *Foxc2* heterozygous mice (Scallan et al., 2021). Thus, these data indicate that the effect of Foxo1 inhibition is dose-dependent, and a threshold amount of inhibition is required to induce valve formation. Furthermore, although AS1842865 treatment showed promising data in rescuing the valve loss in the *Foxc2* heterozygous mice, inhibiting FOXO1 at the organismal level could lead to defects in other organs because FOXO1 is expressed in many cell types besides LECs. Thus, specifically inhibiting or deleting *Foxo1* in LECs using a more clinically translational method needs further investigation.

In addition to AS1842865 treatment, we showed that increased nuclear β-catenin activity completely resolved the valve loss in the *Foxc2* heterozygous mice. However, it does not mean more β-catenin is better. Since the exon 3 of the β-catenin gene is deleted by Cre recombinase, an excessive amount of β-catenin is accumulated in the cells as β-catenin can no longer be degraded in the destruction complex. In our previous study, we administrated three injections of tamoxifen (TM) at P1, P3, and P5 in the *Foxc2* ^*+/CreERT2*^ mice to completely delete *Foxo1*, but the same protocol cannot be used in this study. We discovered that three injections of TM induced severe vessel dilation due to the excessive amount of β-catenin (data not shown). Thus, we reduced the amount of TM with one injection at P1. Our data showed that the mesenteric lymphatic vessels were no longer dilated, and the valves were still induced by the sufficient amount of β-catenin from exon 3 deletion. Therefore, in addition to *Foxo1* deletion, moderately increased β-catenin activity can rescue the valve loss in *Foxc2* heterozygous mice, the mouse model for lymphedema-distichiasis. However, activation of β-catenin at the organismal level is still not desired due to the function of β-catenin in inducing cancer cell proliferation.

The patients of primary lymphedema are susceptible to developing other comorbidities in addition to poor quality of life, such as metabolic disorders and infections, which could be caused due to dysregulated Foxo1 signaling. Elevated levels of Foxo1 have long been implicated in the pathogenesis of metabolic disorders and drug resistance in certain types of cancers, and small molecule inhibitors of Foxo1 have been proposed as a form of pharmacologic treatment (Goto et al., 2008; Pandey et al., 2016). Hence, drug-induced inhibition of Foxo1 can be clinically relevant in the treatment of lymphedema caused by valve defects. In summary, our study helped to better understand how signaling pathways control lymphatic valve formation and how to fine-tune these pathways to regulate valve formation under disease conditions.

## Methods

### Animals

All mouse husbandry and experiments were performed following the guidelines of the University of South Florida (USF) Institution of Animal Care and Use Committee (IACUC). Male and female mice were used for all experiments, and all mouse models were maintained on the C57BL/6J background and obtained through the material transfer agreement (MTA). The *Prox1-GFP* mouse line was from Dr. Young Kwon Hong from the University of South California (USC), LA. The *Foxc2* ^*+/CreERT2*^ mouse line was obtained from Dr. Sathish Srinivasan at the Oklahoma Medical Research Foundation. The *Ctnnb1* ^*lox(ex3)*^ mice were procured with Dr. Makoto Taketo from Kyoto University, Tokyo. The FOXO1 inhibitor, AS1842856, was obtained from EMD Millipore Corporation, United States, and *in vivo* inhibition was achieved by intraperitoneal injections (i.p.) of AS1842856 (10 mg/kg) in wildtype and *Foxc2* ^*+/CreERT2*^ pups every other day from P7 to P21 and AS1842856 (10 mg/kg) every other day from P7 to P11 or P7 to P14 for whole-mount immunostaining. For nuclear *Ctnnb1* activation experiments, tamoxifen (TM) was injected subcutaneously into P1 *Ctnnb1* ^*lox(ex3)*^; *Prox1-GFP*, *Foxc2* ^*+/CreERT2*^; *Prox1-GFP*, and *Foxc2* ^*+/CreERT2*^; *Ctnnb1* ^*lox(ex3)*^; *Prox1-GFP* pups.

### *Ex vivo* analysis of collecting lymphatics and lymphatic valve counting

TM-administered postnatal pups were euthanized using an IACUC-approved method, and the colleting lymphatics were analyzed at the experimental timepoints. Since the small intestine contains the majority of the lymphatic vasculature in the mesentery, collecting lymphatics from the small intestine right before the colon were imaged *in situ* under a Zeiss V16 fluorescent microscope, and the GFP images were analyzed using Fiji (ImageJ). Each lymphatic branch in the small intestine has a defined collecting lymphatic vessel that is connected to the lymph nodes and the pre-collecting lymphatic branches formed next to the gut wall. The segmented line tool in Fiji was used to measure the length of the collecting lymphatic appearing from the lymph node all the way to the branched pre-collecting lymphatics near the gut wall. The multi-point tool in Fiji was used to count the GFP ^high^ valves on the length of the measured collecting vessel. At least four controls and four KO animals were used for every experiment.

### Whole-mount immunostaining

Tissues were collected and fixed in 2% paraformaldehyde (PFA) overnight on an orbital shaker (Belly Dancer, IBI Scientific) at 4℃. The next day, the fixed tissues were washed with phosphate-buffered saline (PBS) three times for 10 min each time, followed by permeabilization in 1X PBS + 0.03% Triton (X-100) (PBST) for 1 h. The tissues were blocked using 3% donkey serum in 1X PBST for 2 h and then incubated overnight with primary antibodies in 1X PBST on an orbital shaker at 4℃. The following day, the tissues were washed with 1X PBST (four times for 10 min each) to remove primary antibodies and then incubated with secondary antibodies in 1X PBST for 90 min at room temperature on an orbital shaker (Belly button, IBI Scientific) after which the tissues were washed with 1X PBST (four times, 10 min each) and incubated with conjugate antibodies in PBST for 60 min at RT. The tissues were then washed with 1X PBST (four times, 10 min each) to remove secondary antibodies and treated with DAPI (Invitrogen) in PBS for 10 min on an orbital shaker at 4℃ for nuclear visualization. The DAPI was washed with PBS (5 min), and the tissues were mounted with ProLong Diamond Antifade Mountant (Invitrogen) onto glass slides (Superfrost Plus Microscope slides, Fisherbrand) and stored at 4℃ until further use. The slides were imaged using the Leica SP8 confocal microscope, and the figures were created using Adobe Photoshop.

### Cell culture and AS treatment

The human dermal lymphatic endothelial cells (hdLECs) purchased from PromoCell (C-12216) were cultured in the EBM-V2 (PromoCell, C-22121) medium on fibronectin (20 μl/well)-coated 6-well plates. hdLECs were treated under four different conditions: DMSO (1 μM), AS1842856 (EMD Millipore Corporation) 0.1 μM, 0.5 μM, and 1 μM for three different time points: 24 h, 48 h, and 72 h for qRT-PCR. For the Western blot, hdLECs were treated with 0.5 μM AS1842856 for 48 h.

### RNA isolation and qRT-PCR

The RNeasy Plus Mini Kit (Qiagen) was used to isolate total RNA from hdLECs in accordance with the manufacturer’s instructions. cDNA synthesis was performed using the Advantage^®^ RT-for-PCR kit (Takara) according to the manufacturer’s guidelines. qPCR was performed in a QuantStudio 6 Real-Time PCR System (Applied Biosystems) with TaqMan^®^ probes (Applied Biosystems, Thermo Fisher), and the Cq value of each gene was normalized to the those of *GAPDH*.

### Cell lysis and Western blot

hdLECs were washed three times with 1X PBS and 1X RIPA buffer, and 100 X phosphatase inhibitor (Pierce^™^, Thermoscientific) was added. A cell scraper was used to scrape the hdLECs off the 6-well plate. hdLECs were pipetted and submerged in the lysis RIPA solution. The lysate was collected in a 1.5-ml protein LoBind tube and incubated for 15 min in ice on an orbital shaker (Belly button, IBI Scientific) for complete lysis. The lysates were then centrifuged for 20 min at 18,300 RPM at 4℃, and the supernatant was collected in a separate Protein LoBind tube without disturbing the pellet. A bicinchoninic acid assay (BCA assay, Pierce^™^, Thermo Fisher) was performed to calculate protein concentration according to manufacturer’s instructions, and 25 μg of the total protein was used to perform the Western blot. The Invitrogen™ mini gel tank apparatus was used to run the Western blot on a 4%–12% Blot™ Bis Tris Plus polyacrylamide gel. Protein transfer to a PVDF membrane was carried out using an iBlot 2 dry blotting system (Lifesciences, Thermo Fisher) under the P0 module. The iBind Flex Western System (Thermo Fisher Scientific) was used to process the mini blots using an incubation time of > 2.5 h, after which the blots were imaged using a Bio-Rad ChemiDoc imaging system.

### Western blot quantification

Quantification analysis of Western blot bands was performed in the following manner: 1) unsaturated Western blot images (.scn) were opened with Image Lab 6.1 (Bio-Rad); 2) using the “lane and bands” tool on the left sidebar of Image Lab, the lanes were defined and adjusted manually; 3) using the “lane and bands” tool, the bands were identified manually with “add” bands option; 4) the “lane profile” tool was used to omit background; 5) the standard and sample lanes were labeled on the “image info” tool; 6) the adjusted volume (Int) value was obtained using the “lane analysis” tool on Image Lab; 7) each protein’s band volume was normalized to the band volume of the housekeeping protein (β-Actin); and 8) relative expression of each protein was calculated by normalizing the band volume of the experimental group (AS 0.5) to its control (DMSO).

### Statistics

The unpaired two-sided Student’s *t*-test was used to determine significant differences between two groups. One-way ANOVA with Tukey’s multiple-comparison test was used to determine significant differences for data with one independent variable. Two-way ANOVA with Tukey’s multiple-comparison test was performed to determine significant differences for data with two independent variables. *p* < 0.05 was considered significant. All data are represented as means ± SEM. GraphPad Prism software (version 6) was used for all statistical analyses and to plot quantitative data.

## Data Availability

The original contributions presented in the study are included in the article/Supplementary Material; further inquiries can be directed to the corresponding author.
